# Pregnancy in congenital heart disease: risk prediction and counselling

**DOI:** 10.1136/heartjnl-2019-314702

**Published:** 2020-07-01

**Authors:** Iris M van Hagen, Jolien W Roos-Hesselink

**Affiliations:** 1 Cardiology, Erasmus Medical Center, Rotterdam, Zuid-Holland, The Netherlands; 2 Cardiology, Maasstad Hospital, Rotterdam, Zuid-Holland, The Netherlands

**Keywords:** congenital heart disease, pregnancy, medical education

## Introduction

Pregnancy is a major life event for almost every woman. However, for women with heart disease pregnancy is associated with additional risks and deserves special attention. The number of pregnancies in women with congenital heart disease has increased over the past decades and is expected to rise further in the coming years.^1^ Physiological changes in the cardiovascular system during pregnancy may bear a risk for those with congenital heart disease who are not able to sufficiently adapt.^2^ Subsequently, heart failure, arrhythmias and worsening of the cardiac condition may complicate pregnancy and expose mother and child to an increased risk of morbidity and mortality. Congenital heart disease is often already diagnosed and treated at the time women start thinking about pregnancy, and hence counselling and risk prediction can be offered. In contrast to acquired heart disease, congenital heart disease bears a relatively low risk of complications during pregnancy. This is partly attributable to good counselling and close follow-up in specialised centres. Dedicated guidelines on pregnancy and heart disease have become available in the past decade, enabling the physician to provide standard and individualised care during pregnancy.^3^ A multidisciplinary ‘pregnancy heart team’ is required to support management of counselling, follow-up and delivery. This review addresses risk stratification and counselling in women with congenital heart disease contemplating pregnancy.

### Cardiovascular physiology during pregnancy

Pregnancy is associated with various physiological adaptions of the cardiovascular system.^4–6^ Cardiac output needs to increase up to 50% during pregnancy, to enable the fetal circulation, and this increase starts already during the first trimester. There is a 30%–40% decrease in vascular resistance. As part of the cardiac output, plasma volume expands in the first and second trimester, followed by an increase in heart rate of around 10%–20%. Delivery further pushes these changes to a temporary maximum. After delivery, large fluid shifts are responsible for a transient volume overload in the first days post partum.

As a consequence of these haemodynamic changes, echocardiographic studies show a clear increase in left ventricular end-diastolic dimensions, while the systolic measurements remain stable.^7^ The subsequent increase in stroke volume leads to a rise of the ventricular outflow tract velocity, and it mimics a hyperkinetic state. The same probably holds for the right ventricle, although less evidence is available. Finally, the expansion of stroke volume and lower afterload influence absolute regurgitation volumes. Regurgitant lesions will therefore hardly be worse during pregnancy.

Hormonal changes influence the integrity of the vessel wall. The structure of the aortic wall may have a subtle weaker composition, which is not of significant importance to healthy women, but may enhance the risk of aortic dissection in women with aortic disease. Furthermore, pregnancy is known for its hypercoagulable state, which is very relevant in those with a mechanical prosthetic heart valve or Fontan circulation.

Pharmacokinetic processes will change during pregnancy, due to the increased plasma volume and total body water, and through changes in absorption, glomerular filtration rate, hepatic metabolism and protein binding activity.^8^ Moreover, drugs may cross the placental border and reach the fetal circulation. The European Medicines Agency (EMA) and US Food and Drug Administration (FDA) provide available evidence for medication during pregnancy, which is helpful when considering or revising drug therapy during pregnancy and breast feeding.

## Risk stratification

**Figure 1:**
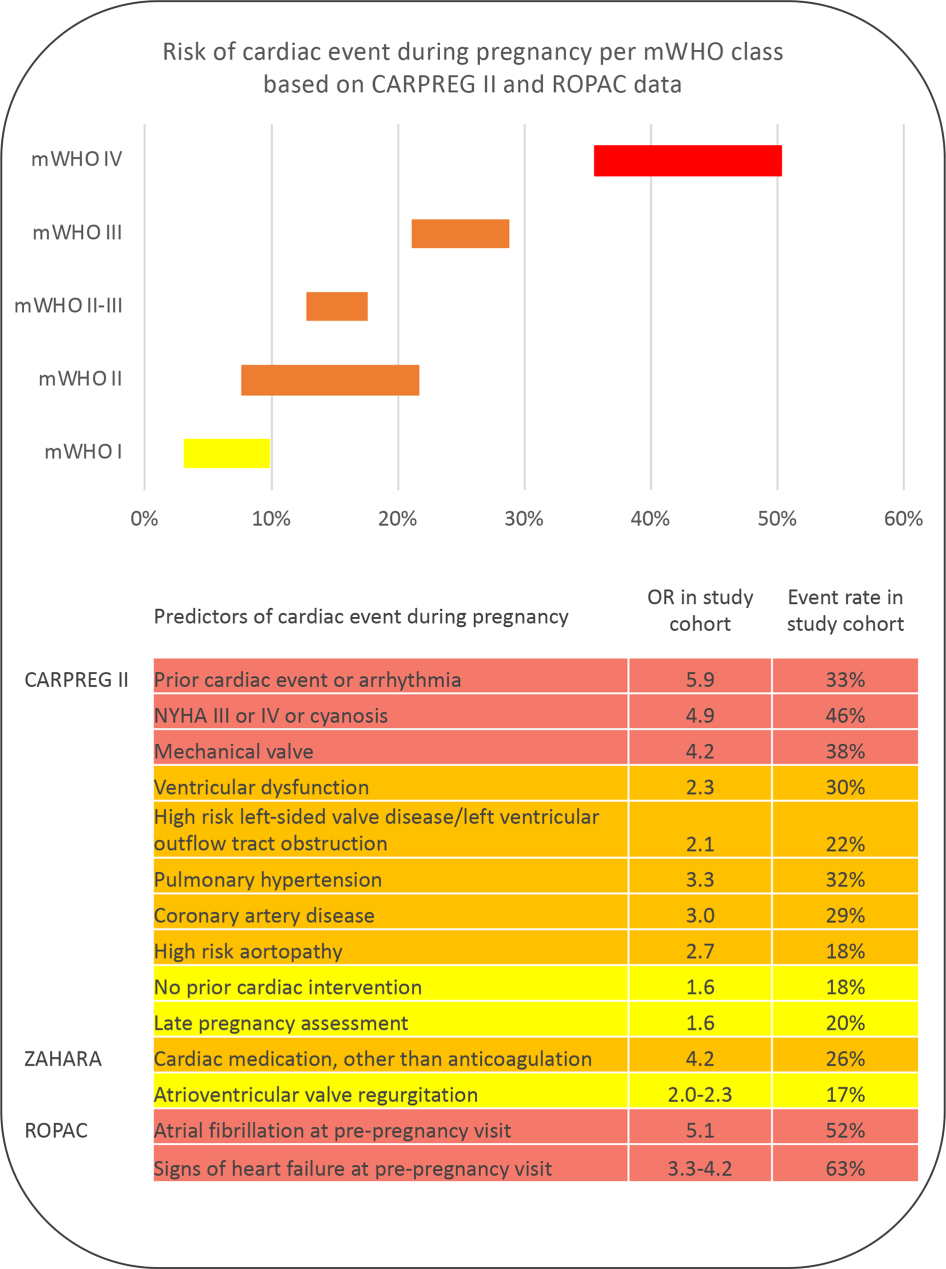
Risk tools modified WHO (mWHO), CARdiac disease in PREGnancy (CARPREG), Zwangerschap bij Aangeboren HARtAfwijking (ZAHARA), Registry Of Pregnancy And Cardiac disease (ROPAC). OR’s and rates are derived from cohorts consisting of approx. 60% patients congenital heart disease (63% in CARPREG II and 58% in ROPAC). Numbers derived from: Silversides et al, JACC, 2018; Drenthen et al, Eur Heart J, 2010; van Hagen et al, Eur J Heart Fail. *NYHA, New York Heart Association functional class. OR, Odds Ratio*

Several risk scores have been proposed to estimate the risk of maternal complications during and after pregnancy (figure 1). The modified WHO (mWHO) risk classification provides an important step in recognition of risks. The stratification is based on the underlying diagnoses and may also give direction as to who should be referred immediately, and who may be evaluated in non-tertiary centres. In addition, two clinical risk tools are available: the CARdiac disease in PREGnancy (CARPREG) and the Zwangerschap bij Aangeboren HARtAfwijking (ZAHARA) risk scores. In women with congenital heart disease, the WHO classification seems to perform best,^9^ but the addition of clinical characteristics will further enable an individualised strategy.

Prepregnancy investigations include thorough history taking, physical examination, an ECG, echocardiogram and exercise test. Clues like diminished exercise tolerance, symptoms of heart failure or palpitations may increase pregnancy risk. Also, a family history of sudden death or dissection is important information to enable risk stratification. Complete physical examination is required to reveal (progression of) heart murmurs, gallop sound, elevated central venous pressure, rales, hepatosplenomegaly and peripheral oedema. Normal pregnancy complaints may be difficult to distinguish from symptoms and signs of heart failure during pregnancy. An ECG provides prognostic information if for instance new or known atrial fibrillation is found. Signs of heart failure or atrial fibrillation before pregnancy are associated with a significant accumulation of complication risk.^10^

Prepregnancy echocardiography should be performed in every woman, with detailed assessment of the cardiac lesion, dimensions, ventricular function and filling pressures. These baseline measurements allow for serial follow-up during pregnancy. Exercise capacity, measuring VO2 max, is an established criterion used in the general evaluation of congenital heart disease, and a sufficient oxygen uptake is associated with better outcome of pregnancy as well.^11^ In individual cases, current cardiac state may be further investigated using other diagnostic modalities such as Holter, cardiac CT or magnetic resonance (CMR) imaging. CT and CMR are used to determine aortic diameters in those with predisposition to or established aortic pathology. Women with aortic disease presenting during pregnancy, without preconception counselling, should preferably be evaluated using CMR without gadolinium, as the effect of gadolinium on the fetus is unknown.^12^ CMR in pregnant women is preferred over CT because of radiation exposure to the fetus.

### Modified WHO risk stratification

**Figure 2:**
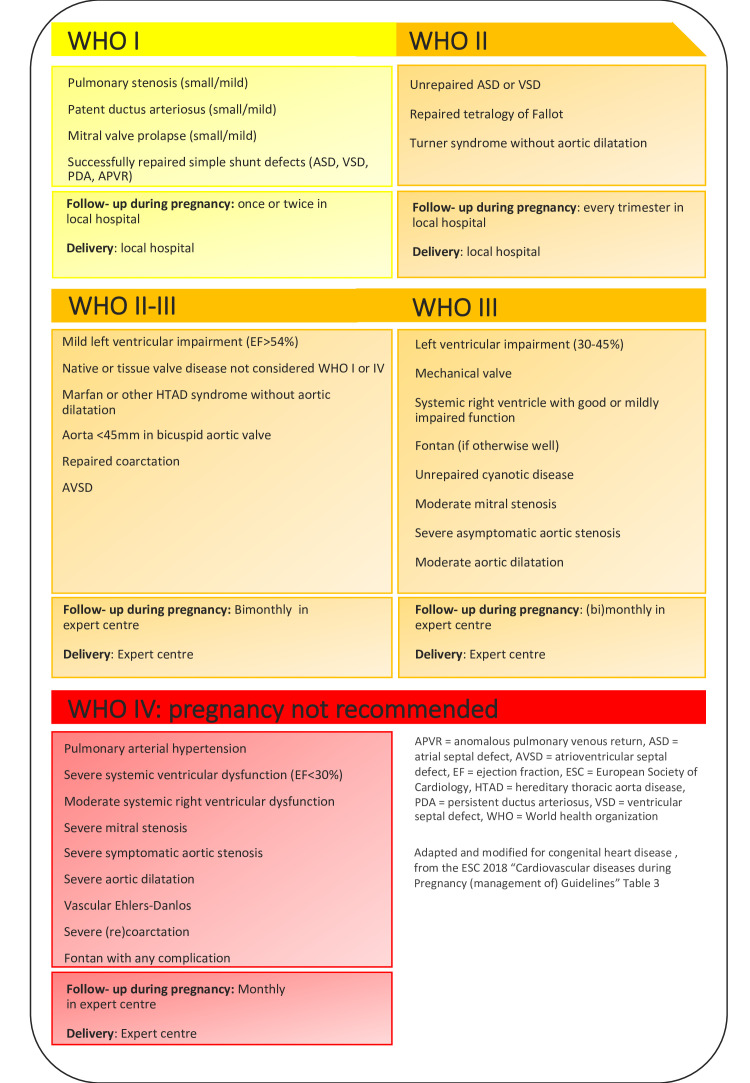
Advised counselling.

The mWHO classification provides a first impression about the potential risk of pregnancy (figures 1 and 2). A comprehensive description of the classification has been published in the latest European Society of Cardiology (ESC) guideline of pregnancy.^3^ Class I consists of mild congenital heart disease such as a small patent ductus arteriosus or mitral valve prolapse, and repaired simple lesions. These lesions are not associated with a significant risk of morbidity or mortality compared with the general pregnant population. The risk of pregnancy gradually increases with an extremely high risk in mWHO class IV, including women with pulmonary arterial dilatation, severe systemic ventricular dysfunction or severe aortic dilatation. Women in class I can be cared for in a peripheral hospital, while those in class IV are advised against pregnancy. The classes mWHO II, II–III and III include women at a mild-to-moderate increased risk, and evaluation and management during pregnancy is required accordingly in a tertiary centre. The mWHO classification is based on expert opinion. It has been tested in several cohorts, and has a moderate discriminative capability in women with congenital heart disease. Therefore, the classification provides mainly a first impression, and more detailed information about pregnancy risks should be obtained through additional clinical information, as included in the risk tools mentioned in the paragraph directly hereafter.

### Clinical risk scores: CARPREG II and ZAHARA

The CARPREG was the first risk tool for pregnancy and heart disease, developed in 2001.^13^ Recently, it was updated and the CARPREG II risk score performed clearly better.^14^ The risk predictors are shown in figure 1 and were derived from, and validated in, a large Canadian cohort of women with heart disease in general, of whom 64% had congenital heart disease. The authors also show the additive information of the CARPREG II predictors on top of the mWHO classification.

The ZAHARA risk score was developed in particular for women with congenital heart disease.^15^ Predictors are listed in figure 1. Two predictors of this risk prediction rule are not mentioned in the CARPREG II study: the use of cardiac medication, and atrioventricular regurgitation.

The Registry Of Pregnancy And Cardiac disease (ROPAC) showed improved accuracy of the WHO classification, by adding prepregnancy atrial fibrillation and signs of heart failure to the classification.^10^

Validation of all tools in different congenital heart disease cohorts lead to different results.^9 16–18^ This is probably driven by several factors. First, not all cohorts focused on congenital heart disease only and congenital heart disease consists of a very broad spectrum of cardiac lesions. Also, centre-specific care and logistic issues such as local infrastructure may influence outcome. Finally, patient-specific factors such as late presentation or patient adherence may hamper the discriminative accuracy. The above-mentioned scoring systems should not be seen as standalone tools: individualised care is crucial based on both type of lesion and clinical features, and evaluated in a multidisciplinary team as discussed hereafter.

## High risk characteristics and congenital defects

Table 1 presents lesion-specific risks for several frequently encountered congenital heart defects. Lesions with high-risk characteristics are discussed in detail in this paragraph. In this section, several specific recommendations are discussed. In general, the ESC guidelines for the management of cardiovascular diseases during pregnancy is a dedicated guideline that can be helpful to all care providers dealing with women with congenital heart disease in childbearing age.

**Table 1:** Lesion-specific risks

	Maternal cardiovascular risk	Obstetric risk (other than caesarean section)	Fetal/Neonatal risk*	References
ASD, repaired	3.6% arrhythmia, 3.6% persistent NYHA deterioration	11% hypertension/pre-eclampsia, 16% PPH	1.8% offspring mortality†	Yap *BJOG* 2009
ASD, unrepaired	4.5% arrhythmia, 3% persistent NYHA deterioration, 0.8% TIA	11% hypertension/pre-eclampsia, 8.3% PPH	3.0% offspring mortality	Yap *BJOG* 2009
VSD, repaired	2.3% arrhythmia	7% hypertension/pre-eclampsia, 12% PPH	21% SGA	Yap *BJOG* 2010
VSD, unrepaired	1% arrhythmia, 1% endocarditis	15% hypertension/pre-eclampsia, 9.6% PPH	6.7% SGA, 1% offspring mortality	Yap *BJOG* 2010
PDA	‡	‡	‡	
AVSD	23% persistent NYHA deterioration, 19% arrhythmias	17% hypertension/pre-eclampsia, 6.3% gestational diabetes, 21% PPH	10% SGA, 6.3% neonatal mortality	Drenthen *EHJ* 2005
TOF	8%–12% arrhythmia or heart failure, 2% persistent NYHA deterioration	8% hypertension/pre-eclampsia, 10% PPH	17%–21% SGA, 18% prematurity, 6.5% offspring mortality	Meijer *Heart* 2005, Balci *AHJ* 2011, Kampman *Us Obst Gyn* 2017
Ebstein	7.3% arrhythmia or heart failure	8.5% PPH	19%–27% prematurity, 18% offspring mortality	Connoly *JACC* 1994, Katsuragi *AJObstGyn* 2013, Lima *Arch Cardiovasc Dis* 2016
LVOT obstruction	3.8%–12% heart failure, 2%–5.7% arrhythmia, 1% endocarditis	6.4%–11% hypertension/pre-eclampsia, 4.2% PPH	8%–21% prematurity, 13% SGA, 0%–1.1% fetal mortality	Silversides *AJC* 2003, Yap *IJC* 2008, Tzemos *AHJ* 2009, Orwat *JACC* 2016
RVOT obstruction	9% heart failure	15% hypertension-related complication	17% prematurity, 4.8% offspring mortality	Drenthen *Heart* 2006, Greutmann *EHJ* 2010
TGA—after arterial switch	0%–12% arrhythmia or heart failure	‡	9%–21% prematurity	Stoll *JAMA* card 2018, Tobler *Am J Car* 2010, Fricke *Heart Lung Circ* 2019, Horiuchi *J Card* 2019
TGA—after atrial repair	6.6%–22% arrhythmia, 11%–14% persistent NYHA deterioration	18% hypertension/pre-eclampsia, 14% PPH	24%–38% prematurity, 22%–38% SGA, 12% offspring mortality	Drenthen *EHJ* 2005, Cataldo *BJOG* 2016, Trigas *Circ J* 2014
ccTGA	26%–32% heart failure, CVA or worsening of cyanosis	2% hypertension/pre-eclampsia, 14% PPH	9% prematurity, 1.3% offspring mortality	Therrien *Am J Card* 1999, Gelson *EJOG* 2011, Drenthen *JACC* 2007
Fontan	3%–37% arrhythmia, 4% thrombotic event, 3%–11% heart failure	14% PPH	59% prematurity, 20% SGA, 7.6%–17% offspring mortality	Garcia Ropero *Circ CV Qual Outcomes* 2018
Cyanotic disease	32% heart failure, arrhythmia or progression of hypoxaemia	10% PPH	37% prematurity, up to 24% fetal mortality	Ladouceur *Circ* 2017, Presbitero *Circ* 1994, Drenthen *JACC* 2007
PAH in CHD (note: very broad spectrum of results)	0%–28% mortality, 31%–35% RV failure, 7% pulmonary hypertensive crisis, 7%–14% thromboembolism, 9% arrhythmia	0%–6% pre-eclampsia, 0%–38% PPH	17%–86% prematurity, 0%–7% offspring mortality	Thomas *JAHA* 2017, Sliwa *EJHF* 2016, Bedard *EHJ* 2009
Eisenmenger	36% mortality, 21%–45% heart failure, 19% thromboembolism	29% PPH	65%–88%% prematurity, 38%–83% SGA, 10%–27% fetal mortality, 18%–25% perinatal mortality	Drenthen *JACC* 2007, Duan *BMC Pregnancy and Childbith* 2016
Coarctation	None reported	22% hypertension/pre-eclampsia	2% offspring mortality	Vriend *EHJ* 2005

This is a generalisation of lesions with very heterogenous patients, included in both prospective and retrospective studies. Specific characteristics such as ventricular dysfunction, valve stenosis or regurgitation, cyanosis and the presence of a mechanical valve may impact risks to a large extent. For detailed information, we recommend to evaluate the papers referred to in the last column. The full references are available in the online additional material (online supplementary file 2).

*SGA and prematurity only reported if >10%.

†Offspring mortality: late fetal death or neonatal death.

‡Not reported.

ASD, atrial septal defect; AVSD, atrioventricular septal defect; ccTGA, congenitally corrected transposition of the great arteries; CHD, congenital heart disease; CVA, cerebrovascular accident; LVOT, left ventricular outflow tract; NYHA, New York Heart Association; PAH, pulmonary arterial hypertension; PDA, persistent ductus arteriosus; PPH, postpartum haemorrhage; RVOT, right ventricular outflow tract; SGA, small for gestational age; TGA, transposition of the great arteries; TIA, transient ischaemic attack; TOF, tetralogy of Fallot; VSD, ventricular septal defect.

10.1136/heartjnl-2019-314702.supp2 Supplementary data

In general, women with characteristics discussed ahead are in modified WHO class III or IV. Women in class IV should be advised against pregnancy following the latest guidelines. However, some women decide to pursue on embarking pregnancy and do return pregnant to the outpatient clinic. Therefore, we will discuss the main issues, and how to perform follow-up in these high-risk situations. Without exception, these women need referral and frequent follow-up in a tertiary centre.

### Pulmonary arterial hypertension

Pulmonary arterial hypertension is indicated as modified WHO class IV. It is associated with a substantial risk of heart failure, ventricular arrhythmias for the mother and also an increased mortality risk, although outcome seems to be better in the current era of advanced therapies. Poor outcome for the fetus is another reason to be very restrained with positive advice on pregnancy. It must be marked that evidence on outcome is limited. Maternal mortality varies from absolute high risk of 28%, to slight improvement in outcome in selected patients with congenital heart disease, but still a maternal mortality rate of 7%.^19 20^ Counselling about the high-risk and thus absolute contraindication to pregnancy remains paramount. When women do become pregnant, options on termination should be given. Otherwise, a plan on strict follow-up, advanced therapy and delivery should be made in a multidisciplinary team with expertise in pulmonary hypertension.

In case of Eisenmenger syndrome, there is also an absolute contraindication for pregnancy. Pregnancy-induced decrease in peripheral vascular resistance causes an additional risk of progressive right-to-left shunt, cyanosis and paradoxical emboli. Very poor fetal outcome can be expected in the majority of cases.^20^

### Cyanosis

Cyanotic disease at adult age may exist in the presence of persistent or uncorrected shunt defects. Early studies showed high numbers of complicated pregnancies in women with cyanotic heart disease. Recently, a retrospective study included 71 pregnancies in 31 women with cyanotic heart disease, without pulmonary arterial hypertension.^21^ Only two women had systemic ventricle dysfunction. Up to 32% of patients developed cardiovascular complications during pregnancy, mainly heart failure and progression of hypoxaemia requiring hospitalisation. Also, late follow-up (with a broad range of 1–15 years) revealed another 13% of chronic heart failure, although natural course may also prompt these late complications. In general, the underlying cardiac lesion will mainly influence maternal risk. The severity of cyanosis confines the chance of a completed pregnancy ending in live birth, with a disappointing low number of live births of only 12% in patients with a saturation below 85%.^22^ Women with a saturation level below 85% are therefore discouraged on embarking pregnancy.

### Fontan

In women with a Fontan circulation, other reasons than cyanosis also influence the level of risk associated with pregnancy. Mainly systemic ventricular dysfunction, and significant atrioventricular valve regurgitation and protein-losing enteropathy are clinical factors associated with higher complication rates. Major complications are heart failure, supraventricular arrhythmias, thromboembolic events and bleeding. Next to these frequent maternal complications, the chance of a pregnancy loss is around 70%.^23^

### Systemic ventricular dysfunction

Any type of congenital heart disease with a diminished ventricular dysfunction is at risk of deterioration during pregnancy. It is an independent risk factor for a complicated pregnancy, as listed by the CARPREG, ZAHARA and ROPAC studies. Signs of heart failure before pregnancy should be treated first as it is a clear additional risk factor. Women with severely diminished left ventricle function, or a moderate systemic right ventricle function should be advised against pregnancy.^3^

### Aortic dilatation

Women with aortic disease may face the risk of further dilatation, or worse, dissection during pregnancy. The extent of diameter growth during pregnancy is difficult to predict and the results diverge between no significant growth up to 3 mm growth during the entire pregnancy with potential decrease of diameter after pregnancy.^24–26^ The risk of dissection depends on the underlying syndrome, as it does outside pregnancy. Hence, women with Marfan syndrome and Loeys-Dietz syndrome are at highest risk. In the absence of Marfan syndrome or other high-risk heritable thoracic aortic disease (HTAD), a cut-off of 50 mm, also in the presence of a bicuspid valve is used to advise against pregnancy. In Marfan syndrome and Loeys-Dietz syndrome or other HTAD, women should not embark on pregnancy in the presence of an aortic root diameter >45 mm.^3^ In Turner syndrome, diameters specifically need to be corrected for body surface area, and a threshold of 27 mm/m^2^ is adopted.^27^ Elective surgery in women beyond these thresholds may be considered, however the risk of type B dissection and other complications is still not zero after surgery. Risk factors such as (family) history of dissection also need to be taken into account.

A caesarean section is advised in all women with an aortic root diameter >45 mm. Below 40 mm, a vaginal delivery is considered safe. Between 40 and 45 mm the choice may depend on diameter growth during pregnancy and risk factors for dissection.

### Mechanical valve

The presence of a mechanical valve is an independent risk factor of complications. The balance between thrombotic and bleeding risks determine the chance of a successful uncomplicated pregnancy, which was about 57% in a large registry of women with a mechanical valve prosthesis.^28^ A valve thrombosis occurred in 4.7%, and 20% of these women died. Anticoagulation strategies are predefined in the ESC guidelines, but a broad spectrum of regimes used globally, emphasises the difficulty of anticoagulation management. In women who are not on low-dose vitamin K antagonists, a switch to some type of heparin in the first trimester is advised, due to the teratogenicity of vitamin K antagonists. To limit the risk of thrombosis with heparin, women should be switched back to a vitamin K antagonist at the start of the second trimester, until the 36th week. A plan for heparin prescription around delivery should be ready. However, still there is no clear consensus on the best anticoagulation regime.^3 29 30^

## Counselling

Prepregnancy counselling is crucial to identify the high-risk patients, and to reassure many patients who are at low risk, because pregnancy is well tolerated in most women with congenital heart disease. During counselling several topics need to be discussed: the risk for the mother and for the fetus, medication use and possible adaptations needed before pregnancy, the recurrence risk for congenital heart disease in the baby and the long-term outcome for the mother. Also reproductive therapies and contraception need attention.

### Maternal and fetal risk

In the largest prospective cohort of 3295 pregnant women with congenital heart disease, mortality occurred in 0.2%, heart failure in 6.2%, an arrhythmia in 2%, a thrombotic event in 1% and aortic dissection in 0.03%.^31^ As discussed, the maternal and fetal risks depend on the underlying disease. In general, the risk of heart failure, arrhythmias and deterioration of cardiac defect should be mentioned during counselling. figure 1 can guide in the emphasis to be made in each individual based on their clinical characteristics. In women with aorta pathology, after risk stratification, the risk of aortic dissection needs discussion.

Obstetric events such as pregnancy-induced hypertension (3%), (pre-)eclampsia (2%) and postpartum haemorrhage (3%) do not occur more often in women with congenital heart disease in general.^31^ Caesarean section is performed in as much as 40% of women, while it is generally preserved for women with an obstetric indication or in a high-risk situation such as heart failure, early labour while on oral anticoagulation or in advanced pulmonary hypertension or Eisenmenger.

Overall, uteroplacental flow is lower in women with congenital heart disease, compared with normal pregnant women. An impaired uteroplacental flow is associated with adverse fetal and neonatal outcome.^32^ As such, fetal growth restriction occurs more often in mothers with a complex congenital heart disease.^33^ Women with congenital heart disease face a higher risk of fetal mortality (1%), premature birth (14%) and a low Apgar score (6%).^31^ Neonatal death occurs in 0.5% and is comparable to the background population, but this depends on the underlying congenital diagnosis of the mother (table 1).

### Assisted reproductive therapies

With the advancing technologies conception also becomes an option for women with congenital heart disease dealing with subfertility or infertility. However, assisted reproductive therapies may be prothrombotic and may induce hypertensive complications, depending on the method and doses used. Overstimulation can lead to ovarian hyperstimulation syndrome. There is an additional risk of conceiving multiple pregnancy which may be poorly tolerated. Thus, women in high-risk conditions such as WHO class III and IV should be discouraged to use these advanced methods if it requires hormonal stimulation, or at least natural cycle options should be considered to prevent the risk of overstimulation.^3^

### Contraception

Women with congenital heart disease are sexually active at about the same age as other women, with a significant amount of unintended pregnancies.^34 35^ Women with a high-risk condition, or those who are advised against pregnancy, need careful advice about contractive methods already at young age. A balanced choice needs to be made between effectiveness, safety and personal preference. Little data are available on safety of contraception in women with congenital heart disease.^36^ Oral contraception may be associated with a potential increased risk of thromboembolic complications, specifically in women with an increased risk of thrombosis, such as a Fontan palliation, although the evidence is contradictory. Thrombosis risk is highest in ethinyloestradiol-containing contraceptives. Progestin-only contraceptives are a suitable alternative. Levonorgestrel-based intrauterine devices are probably safest.^37^ Irreversible options such as tubal occlusion or vasectomy are a serious option to discuss with women in WHO class IV, although there is a general increased operation risk including postoperative infection.

Case reports are available on endocarditis following intrauterine device implantation in specific congenital heart diseases such as Fallot.^38^ Since there is no evidence that the risk of endocarditis is increased compared with the background population, endocarditis prophylaxis is not indicated in genitourinary tract procedures.^39^

### Recurrence risk

Part of the preconception counselling is the risk of recurrence of congenital heart disease in offspring as it is increased. The extent of recurrence risk depends on underlying defect. In autosomal dominant diseases such as Marfan syndrome, the risk is obviously 50%. In the absence of a clear genetic diagnosis, the risk is estimated at 2.9% for all offspring of women with congenital heart disease.^40^ The specified recurrence risks are listed in table 2. In all patients with congenital heart disease, genetic counselling may be considered. Particularly in those with aortic disease, those who have other affected family members or if other non-cardiac congenital abnormalities might be present. In patients with a known genetic defect, preimplantation diagnostic testing has become available.^41^

**Table 2:** Recurrence risk of congenital heart disease

Atrial septal defect	4.5%–6%
Ventricular septal defect	6%–9.5%
Patent ductus arteriosus	4%
Atrioventricular septal defect	7.5%–15%
Ebstein	3.9%–6%
Tetralogy of Fallot	2.5%–10%*
Transposition of the great arteries	0.5%†
Bicuspid aortic valve	4.6%–9.3%
Aortic coarctation	4%
Marfan syndrome	50%
Pulmonary valve stenosis	7%

Modified and updated from van Hagen/Roos-Hesselink, SA Heart 2014.

*Range varies to 50% if associated with 22q11.2 deletion.

†Total recurrence risk, affected mother or father.

### Medication

Medication used before pregnancy should be evaluated for teratogenicity. ACE inhibitors and angiotensin receptor blockers (ARB) have potential adverse effects on the fetus and are therefore contraindicated. In women who are prescribed ACE inhibitors or ARB, a prepregnancy trial without these agents may show whether they remain stable. In women with high risk of heart failure, already pregnant, the risk of discontinuing may outweigh potential fetal risks, a balance to be made by the physician. Beta-blockers can be continued, with strict fetal monitoring because of potential low birth weight. Atenolol is contraindicated during pregnancy, because of reported birth defects. Life-threatening acute heart failure during pregnancy should be treated as outside pregnancy, with no restrictions, to enable the mother to survive such a hazardous event. The FDA classification has been replaced by the Pregnancy and Lactation Labelling Rule, and can be found in prescription labels, and online on the website of both the FDA^42^ and the EMA.^43^ Evaluation of this information is key to enable counselling and provide the best available information on medication during pregnancy.

## Follow-up during pregnancy

Frequency of clinical follow-up depends on risk stratification. Women with a low-risk diagnosis can be seen once or twice in a local hospital. In WHO class II, it is advised to evaluate at least every trimester. If unremarkable than both follow-up and delivery can take place in a local hospital. Women in WHO II-III and higher require follow-up in a dedicated expert centre, and at least bimonthly, increasing per WHO class. Women with cyanosis, pulmonary hypertension or systemic ventricular dysfunction require weekly or biweekly follow-up in the third trimester. Advise on follow-up is summarised in figure 2. A delivery plan should be made in a multidisciplinary team consisting of at least a cardiologist, obstetrician and anaesthesiologist.^3^

The default mode of delivery in almost all women with congenital heart disease is vaginal with spontaneous labour. Exceptions are to be made for obstetric reasons, or in case of a very high-risk cardiac situation as mentioned before. In general, no benefit has been found for caesarean section over vaginal delivery, while gestational age and birth weight in women with a caesarean section is lower.^44^ All women with congenital heart disease should deliver in a hospital and in moderate-to-complex disease in an expert centre.

Additional haemodynamic monitoring during delivery might be required in women who are at risk of acute heart failure or arrhythmias. The first step is pulse oximetry, which includes continuous heart rate monitoring. In advanced risk patients, continuous ECG monitoring should be considered. An arterial catheter for invasive blood pressure monitoring or non-invasive cardiac output measurement provides close follow-up in women who are in WHO class IV, or clinically instable. In suspected high risk of heart failure, consider prolonged observation up to 48 hours after delivery, since this is the timespan of large fluid shifts inducing clinical deterioration.^45^

## Summary

Risk prediction and counselling are the key to limit risks of complications during pregnancy in women with congenital heart disease. The WHO classification and clinical risk tools will guide the physician to the best available risk estimate, but an individualised approach and expert opinion remains paramount in counselling women with congenital heart disease with a pregnancy wish. In women with an estimated low-risk or intermediate-risk pregnancy, planned follow-up and a delivery plan made by a multidisciplinary team provides the best chance of an uncomplicated pregnancy. While the majority do well, there is a small group of women that need an explicit advice not to embark pregnancy, to prevent devastating situations.

**Learning objectives**

- How to estimate risk of pregnancy in women with congenital heart disease.
- What to discuss during prepregnancy counselling in women with congenital heart disease.
- Global overview of follow-up during pregnancy.

**Key messages**

- Prepregnancy prediction of risks for both mother and child can be done based on lesion-specific tools and clinical characteristics.
- The available risk tools all have their limitations, and interpretation needs to be done with care.
- The modified WHO classification is the first step in guiding management and follow-up during pregnancy.
- Clinical characteristics, as provided by the WHO, CARdiac disease in PREGnancy, Zwangerschap bij Aangeboren HARtAfwijking and Registry Of Pregnancy And Cardiac disease studies, and as listed in figure 1, may further define individual risk.
- Counselling should at least consist of explanation of maternal and fetal risks, recurrence risk, evaluation of medication, options for contraception and risks of assisted reproductive therapy.

**CME credits for Education in Heart**

Education in Heart articles are accredited for CME by various providers. To answer the accompanying multiple choice questions (MCQs) and obtain your credits, click on the 'Take the Test' link on the online version of the article. The MCQs are hosted on BMJ Learning. All users must complete a one-time registration on BMJ Learning and subsequently log in on every visit using their username and password to access modules and their CME record. Accreditation is only valid for 2 years from the date of publication. Printable CME certificates are available to users that achieve the minimum pass mark.

10.1136/heartjnl-2019-314702.supp1 Supplementary data
