# Supplementary material for: Pregnancy in congenital heart disease: risk prediction and counselling
Source: Heart. 2020 Jul 1;106(23):1853–61. doi: 10.1136/heartjnl-2019-314702 (PMC7677481; doi:10.1136/heartjnl-2019-314702)
Supplement: Supplementary data [file heartjnl-2019-314702supp002.pdf]

References Table 1

(1-32)

1. Yap SC, Drenthen W, Meijboom FJ, Moons P, Mulder BJ, Vliegen HW, et al. Comparison of pregnancy outcomes in women with repaired versus unrepaired atrial septal defect. BJOG. 2009;116(12):1593-601.
2. Yap SC, Drenthen W, Pieper PG, Moons P, Mulder BJ, Vliegen HW, et al. Pregnancy outcome in women with repaired versus unrepaired isolated ventricular septal defect. BJOG. 2010;117(6):683-9.
3. Drenthen W, Pieper PG, van der Tuuk K, Roos-Hesselink JW, Voors AA, Mostert B, et al. Cardiac complications relating to pregnancy and recurrence of disease in the offspring of women with atrioventricular septal defects. Eur Heart J. 2005;26(23):2581-7.
4. Meijer JM, Pieper PG, Drenthen W, Voors AA, Roos-Hesselink JW, van Dijk AP, et al. Pregnancy, fertility, and recurrence risk in corrected tetralogy of Fallot. Heart. 2005;91(6):801-5.
5. Balci A, Drenthen W, Mulder BJ, Roos-Hesselink JW, Voors AA, Vliegen HW, et al. Pregnancy in women with corrected tetralogy of Fallot: occurrence and predictors of adverse events. Am Heart J. 2011;161(2):307-13.
6. Kampman MA, Siegmund AS, Bilardo CM, van Veldhuisen DJ, Balci A, Oudijk MA, et al. Uteroplacental Doppler flow and pregnancy outcome in women with tetralogy of Fallot. Ultrasound Obstet Gynecol. 2017;49(2):231-9.
7. Connolly HM, Warnes CA. Ebstein's anomaly: outcome of pregnancy. J Am Coll Cardiol. 1994;23(5):1194-8.
8. Katsuragi S, Kamiya C, Yamanaka K, Neki R, Miyoshi T, Iwanaga N, et al. Risk factors for maternal and fetal outcome in pregnancy complicated by Ebstein anomaly. Am J Obstet Gynecol. 2013;209(5):452 e1-6.
9. Lima FV, Koutrolou-Sotiropoulou P, Yen TY, Stergiopoulos K. Clinical characteristics and outcomes in pregnant women with Ebstein anomaly at the time of delivery in the USA: 2003-2012. Arch Cardiovasc Dis. 2016;109(6-7):390-8.
10. Silversides CK, Colman JM, Sermer M, Farine D, Siu SC. Early and intermediate-term outcomes of pregnancy with congenital aortic stenosis. Am J Cardiol. 2003;91(11):1386-9.
11. Yap SC, Drenthen W, Pieper PG, Moons P, Mulder BJ, Mostert B, et al. Risk of complications during pregnancy in women with congenital aortic stenosis. Int J Cardiol. 2008;126(2):240-6.
12. Tzemos N, Silversides CK, Colman JM, Therrien J, Webb GD, Mason J, et al. Late cardiac outcomes after pregnancy in women with congenital aortic stenosis. Am Heart J. 2009;157(3):474-80.
13. Orwat S, Diller GP, van Hagen IM, Schmidt R, Tobler D, Greutmann M, et al. Risk of Pregnancy in Moderate and Severe Aortic Stenosis: From the Multinational ROPAC Registry. J Am Coll Cardiol. 2016;68(16):1727-37.
14. Drenthen W, Pieper PG, Roos-Hesselink JW, Schmidt AC, Mulder BJ, van Dijk AP, et al. Non-cardiac complications during pregnancy in women with isolated congenital pulmonary valvar stenosis. Heart. 2006;92(12):1838-43.
15. Greutmann M, Von Klemperer K, Brooks R, Peebles D, O'Brien P, Walker F. Pregnancy outcome in women with congenital heart disease and residual haemodynamic lesions of the right ventricular outflow tract. Eur Heart J. 2010;31(14):1764-70.

16. Stoll VM, Drury NE, Thorne S, Selman T, Clift P, Chong H, et al. Pregnancy Outcomes in Women With Transposition of the Great Arteries After an Arterial Switch Operation. JAMA Cardiol. 2018;3(11):1119-22.
17. Tobler D, Fernandes SM, Wald RM, Landzberg M, Salehian O, Siu SC, et al. Pregnancy outcomes in women with transposition of the great arteries and arterial switch operation. Am J Cardiol. 2010;106(3):417-20.
18. Fricke TA, Konstantinov IE, Grigg LE, Zentner D. Pregnancy Outcomes in Women After the Arterial Switch Operation. Heart Lung Circ. 2019.
19. Horiuchi C, Kamiya CA, Ohuchi H, Miyoshi T, Tsuritani M, Iwanaga N, et al. Pregnancy outcomes and mid-term prognosis in women after arterial switch operation for dextro-transposition of the great arteries - Tertiary hospital experiences and review of literature. J Cardiol. 2019;73(3):247-54.
20. Drenthen W, Pieper PG, Ploeg M, Voors AA, Roos-Hesselink JW, Mulder BJ, et al. Risk of complications during pregnancy after Senning or Mustard (atrial) repair of complete transposition of the great arteries. Eur Heart J. 2005;26(23):2588-95.
21. Cataldo S, Doohan M, Rice K, Trinder J, Stuart AG, Curtis SL. Pregnancy following Mustard or Senning correction of transposition of the great arteries: a retrospective study. BJOG. 2016;123(5):807-13.
22. Trigas V, Nagdyman N, Pildner von Steinburg S, Oechslin E, Vogt M, Berger F, et al. Pregnancy-related obstetric and cardiologic problems in women after atrial switch operation for transposition of the great arteries. Circ J. 2014;78(2):443-9.
23. Therrien J, Barnes I, Somerville J. Outcome of pregnancy in patients with congenitally corrected transposition of the great arteries. Am J Cardiol. 1999;84(7):820-4.
24. Gelson E, Curry R, Gatzoulis MA, Swan L, Lupton M, Durbridge J, et al. Pregnancy in women with a systemic right ventricle after surgically and congenitally corrected transposition of the great arteries. Eur J Obstet Gynecol Reprod Biol. 2011;155(2):146-9.
25. Drenthen W, Pieper PG, Roos-Hesselink JW, van Lottum WA, Voors AA, Mulder BJ, et al. Outcome of pregnancy in women with congenital heart disease: a literature review. J Am Coll Cardiol. 2007;49(24):2303-11.
26. Garcia Ropero A, Baskar S, Roos Hesselink JW, Girnius A, Zentner D, Swan L, et al. Pregnancy in Women With a Fontan Circulation: A Systematic Review of the Literature. Circ Cardiovasc Qual Outcomes. 2018;11(5):e004575.
27. Ladouceur M, Benoit L, Basquin A, Radojevic J, Hauet Q, Hascoet S, et al. How Pregnancy Impacts Adult Cyanotic Congenital Heart Disease: A Multicenter Observational Study. Circulation. 2017;135(24):2444-7.
28. Thomas E, Yang J, Xu J, Lima FV, Stergiopoulos K. Pulmonary Hypertension and Pregnancy Outcomes: Insights From the National Inpatient Sample. J Am Heart Assoc. 2017;6(10).
29. Sliwa K, van Hagen IM, Budts W, Swan L, Sinagra G, Caruana M, et al. Pulmonary hypertension and pregnancy outcomes: data from the Registry Of Pregnancy and Cardiac Disease (ROPAC) of the European Society of Cardiology. Eur J Heart Fail. 2016;18(9):1119-28.
30. Bedard E, Dimopoulos K, Gatzoulis MA. Has there been any progress made on pregnancy outcomes among women with pulmonary arterial hypertension? Eur Heart J. 2009;30(3):256-65.

31. Duan R, Xu X, Wang X, Yu H, You Y, Liu X, et al. Pregnancy outcome in women with Eisenmenger's syndrome: a case series from west China. BMC Pregnancy Childbirth. 2016;16(1):356.
32. Vriend JW, Drenthen W, Pieper PG, Roos-Hesselink JW, Zwinderman AH, van Veldhuisen DJ, et al. Outcome of pregnancy in patients after repair of aortic coarctation. Eur Heart J. 2005;26(20):2173-8.
