# Supplementary material for: Pregnancy in congenital heart disease: risk prediction and counselling
Source: Heart. 2020 Jul 1;106(23):1853–61. doi: 10.1136/heartjnl-2019-314702 (PMC7677481; doi:10.1136/heartjnl-2019-314702)
Supplement: Supplementary data [file heartjnl-2019-314702supp001.pdf]

6 Multiple choice questions

Q1 Pregnancy is absolutely contraindicated in the following congenital diagnosis, in a patient who feels otherwise clinically well:

1. Cyanotic disease

2. Fontan circulation

3. Eisenmenger syndrome

4. TGA with systemic right ventricle with mild dysfunction

5. Left ventricular dysfunction with an ejection fraction of 40%

Explanation:

All of these diseases carry an increased risk of complications during pregnancy. However, Eisenmenger syndrome is the only cardiac disease of these five, that is classified modified WHO class IV. The risk of maternal mortality is extremely high, estimated at 36% (Table 1), although data is scarce. Heart failure occurs in 21-45%, and poor fetal outcome can be expected.

Q2 A woman with aortic coarctation visits the outpatient clinic for prepregnancy counselling. She asks whether there is a chance that her baby inherits her disease. The recurrence risk of aortic coarctation and other types of congenital heart disease may be estimated at around:

1. 0%

2. 4%

3. 12%

4. 22%

5. 50%

Explanation:

The information can be found in Table 2 and in the next reference: Vriend JW, Drenthen W, Pieper PG, Roos-Hesselink JW, Zwinderman AH, van Veldhuisen DJ, et al. Outcome of pregnancy in patients after repair of aortic coarctation. Eur Heart J. 2005;26(20):2173-8.

Q3 Peripheral vascular resistance during pregnancy decreases

1. 0-10%

2. 10-20%

3. 20-30%

4. 30-40%

5. 40-50%

Explanation:

Cardiac output needs to expand up to 50% during pregnancy. This increase is preceded by a very early decrease in vascular resistance, estimated at 30-40% mainly in the first part (first 20 weeks) of pregnancy. Interesting early data are from Robson et al. Robson SC, Hunter S, Boys RJ, et al. Serial study of factors influencing changes in cardiac output during human pregnancy. Am J Physiol 1989;256:H1060–5.

Q4 A patient with normal aortic diameters and vascular Ehler Danlos contemplates pregnancy. What WHO class would she be in?

1. modified WHO I

2. modified WHO II

3. modified WHO II-III

4. modified WHO III

5. modified WHO IV

Explanation:

Vascular Ehler Danlos is associated with a high risk of aortic dissection. This risk is thought to be further increased during pregnancy. The modified WHO class stratification is shown in Figure 2. Also, an extensive number of diseases and the associated WHO classification is listed in Table 3 of the latest ESC guideline on pregnancy and heart disease. Regitz-Zagrosek V, Roos-Hesselink JW, Bauersachs J, Blomstrom-Lundqvist C, Cifkova R, De Bonis M, et al. 2018 ESC Guidelines for the management of cardiovascular diseases during pregnancy. Eur Heart J. 2018;39(34):3165-241

Q5 A woman presents in the first trimester of pregnancy, while having a mild impaired left ventricular function, but feeling otherwise well. Which of the following drugs is not contraindicated in the first trimester:

1. Metoprolol

2. Warfarin high dose

3. Enalapril

4. Losartan

Explanation:

Metoprolol can be continued during pregnancy. There is a significant chance of lower birth weight, which is why the fetus requires strict and regular follow-up. Note that atenolol is contraindicated, because of reported birth defects. Warfarin is associated with teratogenicity in the first trimester,

and women should be switched to heparin during the first trimester. Fetopathy is the reason that ACE-inhibitors and angiotensin receptor blockers should not be prescribed during pregnancy, and women need consultation on medication switch prior to conception in case of a pregnancy wish.

Q6

In the pregnancy heart team, a delivery plan is discussed for several women. In which diagnosis, a cesarean section is preferred, assuming that there is no obstetric reason and the women are clinically stable:

1. Bicuspid aortic valve and a diameter of 50mm

2. Fontan circulation

3. Pulmonary arterial hypertension

4. Cyanotic disease

5. Systemic ventricle dysfunction

Explanation:

The default mode of delivery in almost all women with congenital heart disease is vaginal with spontaneous labour. Exceptions are to be made for obstetric reasons, or in case of a very high-risk cardiac situation. A cesarean section is advised in all women with an aortic root diameter greater than 45 mm. Below 40mm, a vaginal delivery is considered safe. Between 40-45 mm the choice may depend on diameter growth during pregnancy and risk factors for dissection. These recommendations are based on expert opinion. Regitz-Zagrosek V, Roos-Hesselink JW, Bauersachs J, Blomstrom-Lundqvist C, Cifkova R, De Bonis M, et al. 2018 ESC Guidelines for the management of cardiovascular diseases during pregnancy. Eur Heart J. 2018;39(34):3165-241
